# In Situ Crystalline Growth ZnS Nanoparticles on Conjugated Polymer for Enhancement of the Photocatalytic Performance

**DOI:** 10.3390/polym17050575

**Published:** 2025-02-21

**Authors:** Baotong Liu, Xuelian Li, Long Zhang, Chenghai Ma, Ying Chen, Xinyu Wang, Hongli Wei, Pengfei Wang

**Affiliations:** School of Chemical Engineering, Qinghai University, Xining 810016, China; 15556871893@163.com (B.L.); 15897227469@163.com (X.L.); 13592251891@163.com (L.Z.); 15352962043@163.com (Y.C.); 15829408501@163.com (X.W.); whl517520@163.com (H.W.); wangpfwork@163.com (P.W.)

**Keywords:** sulfur-doped polyimide, zinc sulfide, nanoparticles, Z-type heterojunction, photocatalytic hydrogen production

## Abstract

Photocatalysis is an important means of realizing the efficient use of solar energy and alleviating energy consumption and environmental pollution. This work used a simple solvothermal synthesis method to prepare a series of zinc sulfide/sulfur-doped polyimide (ZnS/SPI) direct Z-type heterostructured photocatalysts. ZnS/SPI heterostructured photocatalysts have better photogenerated electron–hole pairs separation and wider visible light absorption region. The effect of ZnS on the properties of ZnS/SPI composites, such as morphology, structure, and optoelectronic properties, was systematically investigated by a series of characterization tests. These results showed that the photocatalytic activity of the ZnS/SPI composite was significantly improved compared with SPI. The 10ZnS/SPI composite exhibited the highest photocatalytic hydrogen production rate under full irradiation (216.9 µmol/g/h), which was about 2.8 times higher than that of SPI (76.6 µmol/g/h). Moreover, it has a high stability over a long period in the photocatalytic process. The enhanced photocatalytic performance of the ZnS/SPI heterojunction is mainly due to the close contact between the ZnS nanoparticles and the SPI interface, which improves the charge separation and reduces the complexation rate of electron–hole. This work shows that the formation of ZnS/SPI composites Z-type heterojunction can effectively enhance the activity of polymer photocatalysts.

## 1. Introduction

In the context of accelerating energy depletion and environmental deterioration, new energy development and environmental governance have become a hot spot of common concern for countries around the world [1]. Photocatalytic technology has important potential applications in energy conversion and environmental purification. As a kind of green technology, the system exhibits dual functions: degrading organic pollutants in water [2] and generating hydrogen energy through water decomposition [3]. These excellent performances can be well utilized to create sustainable green energy by using solar energy, so it has attracted the attention of researchers [4,5]. The groundbreaking discovery regarding water splitting via single-crystal TiO_2_ photocatalysis was initially revealed through Fujishima and Honda’s research in 1972 [6]. Later, some scholars successfully realized the photocatalytic degradation of organic matter by utilizing the photocatalytic properties of TiO_2_. Since then, photocatalytic technology has been expected to improve the problems existing in energy and the environment [7]. In recent years, the graphitic phase carbon nitride (g-C_3_N_4_), as a novel organic semiconductor photocatalytic material, has been highly efficient in the degradation of organic pollutants and the decomposition of H_2_O to produce H_2_ [8]. The study of photocatalysts has gradually shifted from metal–inorganic semiconductor materials to organic polymers. More and more studies have been conducted on g-C_3_N_4_, which has been widely used in the photocatalytic decomposition of H_2_O to produce hydrogen [9], the degradation of organic matter [10], and the carbon dioxide reduction [11].

π-conjugated polyimide (PI), as another organic semiconductor photocatalytic material, was first reported by Wang et al. in 2012 [12]. Based on PI and g-C_3_N_4_ having a similar structure, rich sources, low price, good chemical stability, and light absorption performance, the structure can be adjusted and environmentally friendly [13]. However, PI still has some defects as a photocatalyst, such as a limited light absorption range and low photocatalytic activity. Therefore, Wang et al. [12] successfully synthesized sulfur-doped polyimide (SPI) by using S_4_ as a dopant and applying in situ doping thermal polymerization method. In which, sulfur (S) atoms replaced the N atoms in the triazine ring of PI, which lowered the bandgap of PI, broadened the absorption of visible light by PI, and strengthened the oxidizability of its holes. Although PI modification strategies have enhanced SPI photocatalytic capabilities, the rapid recombination kinetics of photogenerated charge carriers continue to impede its practical implementation [14,15,16]. To optimize the photocatalytic efficiency of SPI systems, controlling the morphology of photocatalysts as well as constructing inorganic–organic heterojunction structures are commonly used as effective means [7,9,17].

Zinc sulfide (ZnS), as a common semiconductor photocatalyst, has a band gap of about 3.34 eV [18,19]. As an n-type semiconductor material, ZnS is often used to construct heterojunctions with g-C_3_N_4_ [20], which can effectively improve the efficiency and performance of photocatalytic reactions and expand its application areas. In addition, ZnS has the advantages of chemical stability and environmental friendliness for various photocatalytic applications [21,22]. Upon the examination of the available scientific literature, based on the current literature, there are no relevant reports on the combination of ZnS and SPI to construct heterojunctions. Moreover, there is a certain foundation for the study of ZnS combining with g-C_3_N_4_ to construct heterojunction [23,24,25]. For example, Zhu et al. [26] controlled the band gap by doping phosphorus in g-C_3_N_4_, then made it form a heterojunction with ZnS, and finally synthesized PCN/ZnS photocatalysts with enhanced photocatalytic activity.

Based on the above analysis, ZnS/SPI composite photocatalysts were prepared by an in situ crystalline growth method for the first time in this study. ZnS nanoparticles were grown on the surface of SPI by in situ crystallization to form a direct Z-type heterojunction structure for the photocatalytic decomposition of aqueous hydrogen, as well as the degradation of organic pollutants.

## 2. Experimental Section

### 2.1. Chemicals and Materials

Melamine (MA), pyromellitic dianhydride (PMDA) and polyvinylpyrrolidone (PVP, K = 23–27) were purchased from Shanghai Macklin Biochemical Co., Ltd. in Shanghai, China. and sublimed sulfur (S_4_) was bought from Tianjin Dengke Chemical Reagent Co., Ltd. in Tianjin, China. Zinc acetate dihydrate (C_4_H_6_O_4_Zn·2H_2_O) and thioacetamide (TAA) purchased from West Asia Chemical Technology (Shandong) Co., Ltd.in Zibo City, China. Triethanolamine (TEOA) was obtained from Sigma-Aldrich in St. Louis, MO, USA. All chemical reagents have a purity greater than 99.0% and were used without further purification.

### 2.2. Synthesis of the Photocatalyst

The preparation of sulfur-incorporated polyimide materials was accomplished via a facile thermal polymerization approach in the solid state, following established protocols documented in previous publications [12]. Usually, 1.0 g of melamine (MA), 0.9 g of sulfur and 1.7 g of phthalic tetra-dicarboxylic anhydride (PMDA) are weighed and ground in the same mortar, so that the reactants are fine and homogeneous particles are loaded into a small ceramic boat, and then put into a tube furnace. Nitrogen was passed through the mouth of the tube after assembling and sealing the tube to prevent the sulfur element from being oxidized. It was heated to 325 °C in a nitrogen atmosphere at a ramping rate of 2.5 °C min^−1^ and held for 240 min. Then, it was cooled to room temperature at a cooling rate of 2.5 °C min^−1^. The raw SPI product subsequently underwent a series of post-treatment steps, including washing with water at 50 °C while stirring for 30 min, followed by filtration. During the filtration process, the sample was washed three more times with water. After the water was removed by filtration, the sample was dried in an oven at 60 °C for 8 h. Finally, the dried sample was ground into a powder using a mortar to obtain the target SPI photocatalyst.

**Scheme 1:**
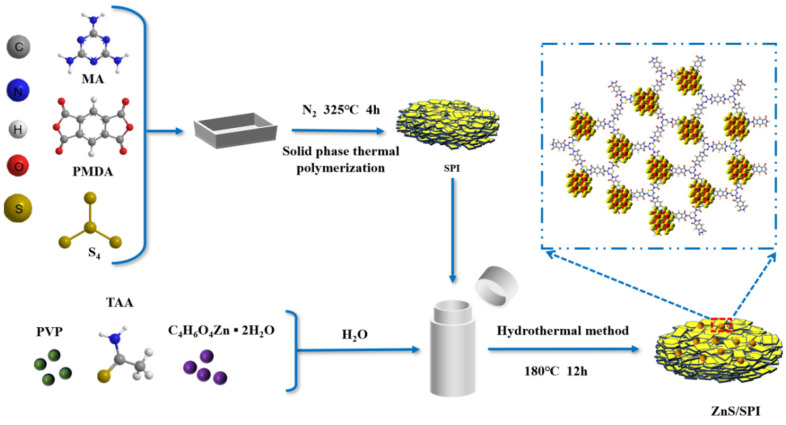
Preparation process diagram of ZnS/SPI composite.

ZnS/SPI composites were synthesized by an in situ crystalline growth method. The prepared SPI was dissolved with zinc acetate dihydrate, thioacetamide, and polyvinylpyrrolidone (PVP) according to a certain ratio with an appropriate amount of ultrapure water and dispersed ultrasonically using an ultrasonic cleaner for 10 min. It was transferred to a magnetic stirrer at 80 °C and stirred into a suspension. Then, it was heated up to 180 °C at a rate of 2.5 °C/min in a reactor to carry out a hydrothermal reaction for 12 h. Finally, the crude product was further washed, dried, and milled. Based on the theoretically calculated ZnS mass ratios, a graded series of ZnS/SPI composite photocatalysts with precisely controlled loadings (3, 7, 10, 14, 17, and 20 wt.%) were fabricated via hydrothermal synthesis. For convenience, they were referred to as 3ZnS/SPI, 7ZnS/SPI, 10ZnS/SPI, 14ZnS/SPI, 17ZnS/SPI, 20ZnS/SPI, respectively. The amount of reactants used to synthesize ZnS/SPI samples is indicated in Table S1. The preparation process of ZnS/SPI composites is shown in Scheme 1.

Zinc sulfide (ZnS) was prepared in the same manner as ZnS/SPI, but without the introduction of SPI during the preparation process, resulting in pure zinc sulfide.

### 2.3. Catalyst Characterization

Structural characterization was implemented via powder X-ray diffraction (PXRD) measurements utilizing Cu Kα radiation on a D/max2500PC diffractometer (Rigaku Corporation, Tokyo, Japan.) operated at 40 kV/40 mA. Surface morphology examination was accomplished using an MIRA LMS field-emission scanning electron microscope (TTESCAN Brno, Czech Republic). Detailed microstructural analysis, including transmission electron microscopy (TEM), high-resolution TEM (HRTEM), and selected area electron diffraction (SAED), were performed on the JEM-2100 instrument produced by Akishima JEOL Ltd. in Tokyo, Japan. Chemical bonding information was acquired through Fourier transform infrared (FT-IR) spectroscopy on a Nicolet 6700 system (Thermo Fisher Scientific, Inc., Waltham, MA, USA) with samples prepared as KBr pellets. Surface electronic states were investigated by X-ray photoelectron spectroscopy (XPS) and valence band XPS (VBXPS) using a PHI 5000 Versa Probe system (ULVAC-PHI, Inc., Chigasaki, Japan) with monochromatic Al Kα radiation, calibrating all binding energies against the C 1s reference (284.6 eV). Optical properties were evaluated through UV–visible diffuse reflectance spectroscopy (DRS) using a UV-2600 spectrophotometer (Shimadzu, Tokyo, Japan) with BaSO_4_ as the reference material at ambient temperature. Photoluminescence (PL) measurements were conducted on an F-7000 fluorescence spectrometer (Hitachi High-Tech Corporation, Tokyo, Japan) with excitation at 350 nm. The transient fluorescence spectra of the photo-catalysts were recorded by an FLS980 multi-function steady-state and transient fluorescence spectrometer (Edinburgh Instruments, Livingston, UK) at room temperature. The average lifetime <τ_avg_> was calculated by using the equation: $\tau_{\mathrm{avg}}=\frac{A_{1}\tau_{1}^{2}+A_{2}\tau_{2}^{2}}{A_{1}\tau_{1}+A_{2}\tau_{2}}$, $\;{\;\tau }_{\mathrm{avg}}=\frac{A_{1}\tau_{1}^{2}+A_{2}\tau_{2}^{2}+A_{3}\tau_{3}^{2}}{A_{1}\tau_{1}+A_{2}\tau_{2}+A_{3}\tau_{3}}$ [27]. Electron spin resonance (ESR) analysis was executed using an EMXplus spectrometer (Bruker, Billerica, MA, USA).

### 2.4. Electrochemical Tests

The photoelectrochemical (PEC) characterization was conducted on a CHI 660 D potentiostat/galvanostat (Chenhua Instrument Co., Ltd., Shanghai, China) equipped with a conventional three-electrode configuration and illuminated by a Xe lamp source. The electrochemical cell comprised an Ag/AgCl reference electrode, a platinum counter electrode, and a sample-modified conductive glass serving as the working electrode. A neutral aqueous solution of Na_2_SO_4_ (0.5 M, pH 6.8) functioned as the supporting electrolyte. Fluorine-doped tin oxide (FTO) transparent conductive glass was used to prepare the working electrode. In a typical preparation procedure, the catalyst sample (5 mg) was suspended in anhydrous ethanol solution (1 mL) to form suspension A, while Nafion solution (20 μL) was homogeneously mixed with anhydrous ethanol (1 mL) to obtain suspension B. Subsequently, both suspensions underwent ultrasonic treatment for 30 min. Subsequently, on the 1 × 2 conductive glass coating (coating to the 1/2 position), the first is 50 μL A at 10 μL each time to coat five times to the conductive glass, each time after the coating to dry at a certain temperature for 1–2 min, to ensure that the sample adheres to the conductive glass, and then coated with 20 μL B at 10 μL each time to coat two times (to be coated on the conductive side). Mott–Schottky curves were measured at 1.0 kHz under dark conditions. Electrochemical impedance spectra (EIS) plots were collected in the frequency range of 200 kHz–10 MHz.

### 2.5. Photocatalytic Performance Test

Bofulai Technology Co., Ltd., Beijing, China. XL-300W xenon lamp (I = 20A) was used as the degradation light source, together with a filter (λ > 420 nm) to simulate visible light, The photocatalytic performance of the as-prepared materials was evaluated by monitoring the degradation efficiency towards methyl orange (MO) as a model organic contaminant. In a typical procedure, the photocatalytic system was established by introducing 200 mg of the as-prepared catalyst into a reactor containing 100 mL methyl orange solution (40 mg/L). The reaction mixture was maintained under dark conditions with continuous magnetic agitation in a temperature-controlled circulating bath for 1 h to achieve adsorption–desorption equilibrium. Subsequently, an aliquot (3–4 mL) of the homogeneous suspension was withdrawn for analysis. A xenon lamp (300 W) was switched on for photocatalytic degradation, and mixed suspensions (3−4 mL) were withdrawn as samples at 1 h intervals and filtered through MCE 0.45 μm filters, followed by the measurement of absorbance at 464 nm using a UV–visible spectrophotometer (Mapada UV-1800 is from Mapada Instruments Co., Shanghai, China, Ltd.). The catalyst samples were tested for photocatalytic hydrogen production activity in a top-illuminated reactor using an XL-300 W xenon lamp (Bofulai Technology Co., Ltd., Beijing, China) as a fully irradiated light source. Typically, 50 mg of photocatalyst was dispersed in a mixed solution containing 100 mL of triethanolamine (CTOA) cavity sacrificer/water (1:9 *v*/*v*), to which the hydrogen generation was determined by gas chromatography (GC9790II, Fuli Instruments is from Zhejiang Fuli Analytical Instruments Co., Ltd. in Taizhou, China. TCD, Ar carrier) by adding an appropriate amount of H_2_PtCl_6_ as the precursor of Pt, irradiated with fully irradiated light (λ > 300 nm) for 2 h, and using 3 wt% of Pt as the co-catalyst to determine the hydrogen generation.

## 3. Results and Discussion

### 3.1. Structure and Morphology Analysis

**Figure 1:**
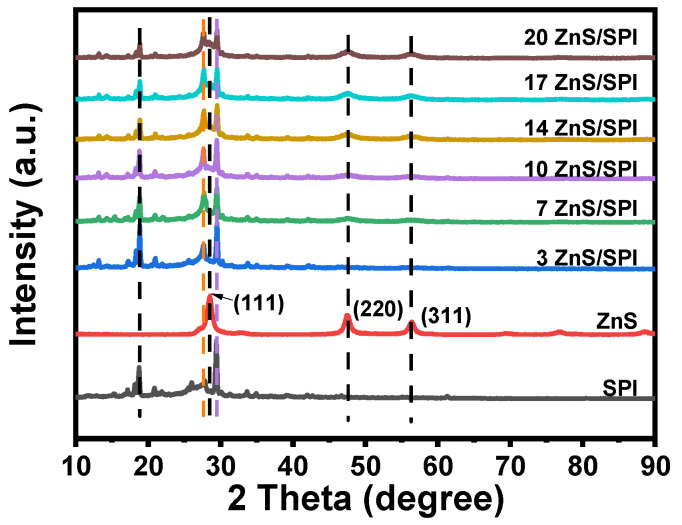
XRD patterns of SPI, ZnS, and ZnS/SPI composites.

Structural investigation of crystallographic features and phase identification was conducted via powder X-ray diffractometry (XRD) measurements. Figure 1 illustrates the diffraction profiles obtained from synthesized SPI, ZnS, and their hybrid ZnS/SPI nanocomposites. The XRD pattern reveals characteristic diffraction peaks of SPI located within the 10–30° region, aligning with previously documented SPI diffraction patterns in the literature [12]. For pure ZnS crystals, Diffraction signals appearing at 28.5°, 47.5°, and 56.4° are indexed to the (111), (220), and (311) crystallographic planes, characteristic of the β-ZnS phase [28]. In addition, there were no other diffraction peaks, which indicated that all precursors (zinc acetate dihydrate) had been completely converted to ZnS. Moreover, it is more clear that ZnS has relatively sharp characteristic peaks, which indicates high purity and high crystallinity, suggesting that ZnS synthesized by solvothermal synthesis agrees with the literature [29,30].

For the ZnS/SPI composite samples, the diffraction peaks unique to SPI and the characteristic peaks of ZnS can be observed from them, and their positions have not been changed, which indicates that the in situ crystalline growth of the ZnS nanoparticles on the surface of SPI using solvothermal synthesis. Moreover, it has not changed the native crystalline architecture and polymeric sequence of SPI. Notably, when comparing the ZnS/SPI composite photocatalysts with pristine SPI, a substantial reduction in the diffraction intensity was observed at 29.5°. On the contrary, the peak intensity of the π-conjugated two-dimensional (2D) frames stacked at 27.4° of SPI was enhanced with the increase in ZnS content [8]. This may be because, throughout the synthetic process of ZnS/SPI hybrid materials, some of the polymers on the surface layer of SPI were stripped into oligomeric fragments under the conditions of intense stirring and secondary roasting (1400 r/min, 180 °C), which led to a weakening of the peaks of the crystal surface with high crystallinity (29.5°). In turn, the exfoliated fragments are gradually stacked onto the low crystallinity (27.4°) crystal surface. Analogous observations have been documented in prior research studies [15]. In addition, by comparing the XRD patterns of SPI, ZnS, and ZnS/SPI composites, the characteristic peaks of ZnS in ZnS/SPI are almost absent, this phenomenon can be attributed to both the limited quantity and uniform distribution of ZnS throughout the matrix. With the increase in ZnS content, the characteristic peaks of ZnS began to gradually enhance, while the characteristic peak locations exhibited consistent patterns throughout the analysis, which also further indicated that the composites prepared by the in situ polymerization method did crystallize ZnS growth on the surface of SPI, which was similar to our previous study [31].

**Figure 2:**
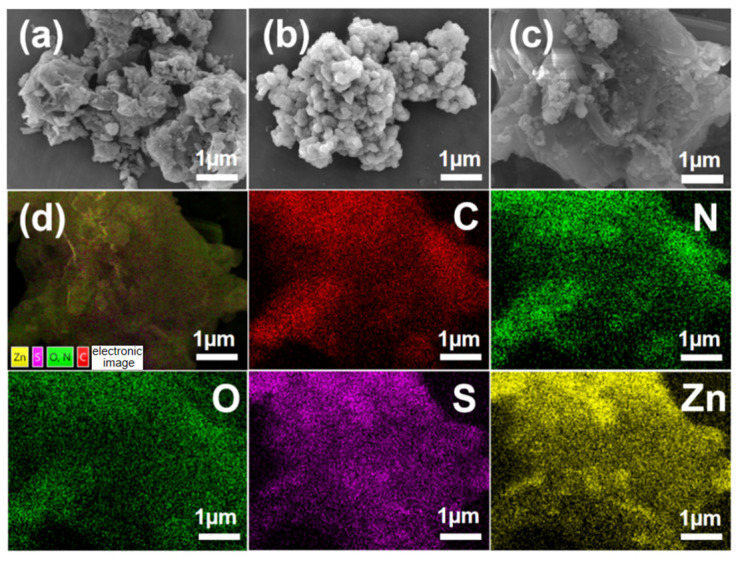
(**a**–**c**) SEM images of SPI, ZnS, and 14ZnS/SPI; (**d**) EDS mapping of C, N, O, S, and Zn elements for 14ZnS/SPI samples.

The morphological characteristics and structural features of synthesized SPI, ZnS, and 14ZnS/SPI hybrid materials were examined using scanning electron microscopy (SEM), as illustrated in Figure 2. SPI is a loosely stacked irregular lamellar morphology with irregular pores [12]. And ZnS shows the form of nanoparticles. The 14ZnS/SPI samples indicated ZnS nanoparticles combined with SPI nanosheets. These findings corroborate the results obtained from XRD structural analysis. In addition, the extensive interfacial interaction established between the SPI matrix and ZnS nanoscale particles facilitated the efficient transport of photo-induced electrons toward the hybrid material’s surface [14]. However, the ZnS nanoscale particles exhibited substantial aggregation, manifesting distinct block-like clusters of accumulated structures. The severe agglomeration of the nanoparticles obscured the active sites of the ZnS catalyst. Therefore, the incorporation of SPI effectively promotes the uniform distribution of ZnS crystallites, preventing particle coalescence [32]. This is more favorable with charge carrier transport, thus contributing to improved photocatalytic activity. In addition, to elucidate the spatial arrangement of C, N, O, S, and Zn components within the 14ZnS/SPI framework, elemental distribution analysis was conducted using SEM mapping techniques. As illustrated in Figure 2c, all elements are uniformly distributed in the prepared photocatalyst skeleton. These results indicate that ZnS has been loaded on the surface of SPI.

**Figure 3:**
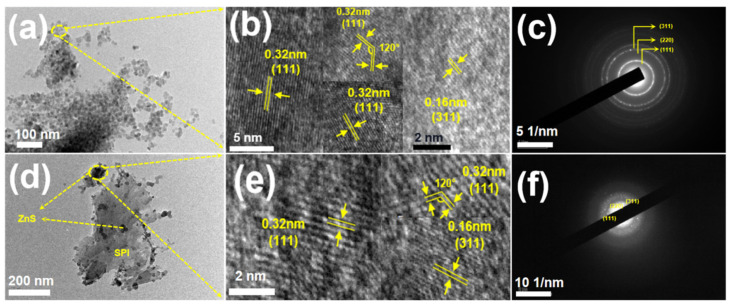
The images of (**a**) TEM, (**b**) HR-TEM, and (**c**) selected area electron diffraction (SAED) patterns for ZnS, and the images of (**d**) TEM, (**e**) HR-TEM, and (**f**) SAED patterns for 14ZnS/SPI composite.

To further investigate the internal morphology of ZnS and 14ZnS/SPI, high-frequency transmission electron microscopy was used to analyze the morphology of the prepared ZnS and 14ZnS/SPI. Figure 3a clearly shows the morphology of ZnS nanoparticles, which is consistent with the SEM morphology. The transmission electron microscopy analysis in Figure 3d reveals the characteristic laminar architecture of the 14ZnS/SPI composite materials. Figure 3b,e show the lattice diffraction fringes of ZnS and 14ZnS/SPI composites. As revealed in Figure 3b, analysis of the crystallographic structure demonstrated characteristic interplanar distances of 0.32 nm and 0.16 nm, which can be indexed to the (111) and (311) crystallographic planes, respectively [26]. Meanwhile, the selected area electron diffraction (SAED) pattern presented in Figure 3c demonstrates the predominant crystallographic planes of ZnS corresponding to (111), (220), and (311) indices [29]. These crystallographic observations correlate precisely with the XRD diffraction patterns of ZnS presented in Figure 1. The structural analysis depicted in Figure 3e, the SAED image of 14ZnS/SPI shows well-defined boundaries between the ZnS particles and the SPI lamellae, and the SPI is embedded with many ZnS with clear lattice stripes. Where the lattice stripe spacing is consistent with that described in 3b above. Lattice fringes of SPI were not observed in this study due to in-plane diffraction [33]. Meanwhile, the SAED images of the 14ZnS/SPI composite further illustrate ZnS with its major (111), (220), and (311) crystalline facets as shown in Figure 3f.

**Figure 4:**
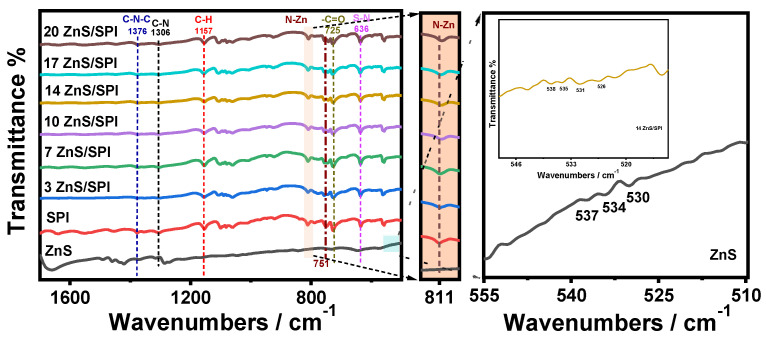
FT−IR patterns of ZnS, SPI, and ZnS/SPI composite powder samples.

Fourier transform infrared (FTIR) spectroscopic characterization of ZnS, SPI, and ZnS/SPI composite powders is presented in Figure 4. Detailed spectral analysis reveals that the ZnS/SPI composites exhibit predominant vibrational modes characteristic of the SPI component, with distinctive IR absorption bands maintained in the composite structure. In agreement with XRD analysis, the FTIR spectroscopy revealed that the ZnS/SPI composites exhibited significantly attenuated absorption intensities relative to pristine SPI. The characteristic vibrational signatures at 1725 and 725 cm^−1^ were unambiguously assigned to the symmetric stretching and bending modes of the carbonyl (−C=O) functionalities residing in the PMDA moiety of SPI, respectively [12,34]. Absorption bands located at 1560 cm^−1^ and 1306 cm^−1^ marked with black dashed lines indicate the C−N−C stretching vibration in the five-membered imide ring and the aromatic C=N respiration mode in the triazine ring, respectively [35,36]. The addition of ZnS attenuated these two absorption peaks and these results may be due to the strong interaction between ZnS and SPI. The peak marked with a blue dashed line at 1376 cm^−1^ corresponds to the characteristic vibrational absorption peak of the C−N−C bond in the five-membered imide ring [37]. The absorption peaks marked with red dashed lines at 1640 and 1157 cm^−1^ are then the characteristic vibrational absorption peaks of the C−C and C−H bonds in the aromatic ring of the anhydride portion [38]. The position of the ZnS/SPI composite peaks is almost unchanged compared to SPI, attributed to the minimal loading and uniform distribution of ZnS across the SPI surface.

In addition, a distinctive spectral signature, denoted by a purple dashed line, appears at 636 cm^−1^, which corresponds to the fundamental vibrational mode of the sulfur–nitrogen (S−N) linkage. The spectral analysis reveals the characteristic signals of ZnS within the region of 525−540 cm^−1^ are found in the composites of 14ZnS/SPI, confirming the presence of ZnS. No new absorption peaks appeared in the 14ZnS/SPI system, but some of the absorption peaks underwent a significant displacement, which confirms the existence of the interaction between ZnS and SPI. Meanwhile, the analysis confirms that the structural integrity of SPI remained unaltered following the introduction of zinc sulfide nanoparticles, but formed Zn−N coordination bonds on its intersurface, which was conducive to the improvement of the photocatalytic stability of the composites [14]. It can also be seen from the figure that SPI has an absorption peak at 751 cm^−1^, but changes to two consecutive weak peaks in the composite, which is speculated to be due to the strong interaction between SPI and ZnS. A characteristic shift in the vibrational frequency from 811 cm^−1^ (pure SPI) to 808 cm^−1^ (composite) is observed, suggesting the establishment of coordinative Zn−N interactions. This phenomenon arises from the electronic coupling between the unfilled d-orbitals of zinc atoms and the electron-rich sp^3^ hybridized nitrogen centers, leading to a redistribution of electron density where zinc atoms become more electron-abundant while nitrogen atoms experience reduced electron density [39]. The presence of a strong chemical bonding between ZnS and SPI is verified. This robust interaction enhances the transfer of photogenerated charge carriers between ZnS and SPI through their intimate interface.

**Figure 5:**
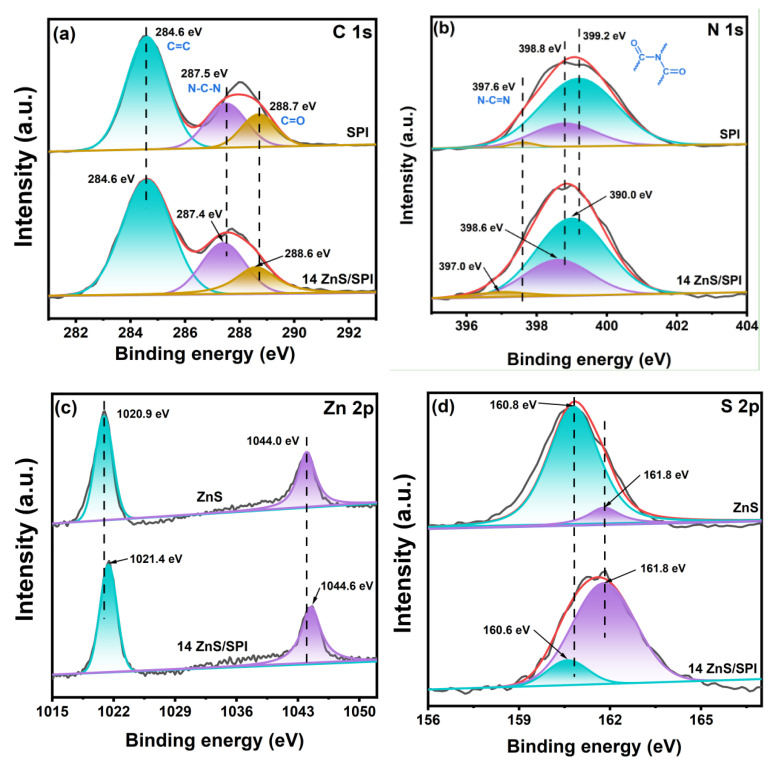
XPS spectra for (**a**) C 1s, (**b**) N 1s, (**c**) Zn 2p, and (**d**) S 2p of SPI, ZnS, and 14ZnS/SPI composite.

X-ray photoelectron spectroscopy (XPS) measurements were employed to elucidate the elemental composition and chemical bonding states at the surface interfaces of pristine ZnS, unmodified SPI, and the synthesized 14ZnS/SPI composite system. And, the binding energy of each element was corrected using the C1s peak (284.6 eV). In addition the high-resolution spectra of C 1s, N 1s, Zn 2p, and S 2p are shown in Figure 5. The high-resolution C 1s XPS spectrum of SPI is shown in Figure 5a, where three peaks at 284.6 eV, 287.5 eV, and 288.7 eV can be observed, corresponding to sp^2^ C=C and N−C−N and C=O in the triazine ring in SPI, respectively [33]. The three characteristic peaks at 397.6 eV, 398.8 eV, and 399.2 eV in the N 1s XPS spectra of the SPI in Figure 5b are mainly attributed to the N−C=N bonding on the triazine ring of SPI, as well as the splitting of the N atoms in the five-membered imide ring of the polyimide, the polyimide, after the supersulfuration [26]. In Figure 5d, the high-resolution S 2p XPS spectrum of ZnS shows two peaks at 160.8 eV and 161.8 eV attributed to the basal sulfur ions (S^2−^) and the S 2p_1/2_ of the apical S^2−^ in ZnS, respectively [31]. In Figure 5c, the Zn 2p XPS spectrum of ZnS shows two peaks at 1020.9 eV and 1044.0 eV attributed to the Zn 2p_1/2_ and Zn 2p_3/2_ states of Zn^2+^, respectively [19]. SPI complexed with ZnS revealed a significant displacement of the peak positions of the elements in the samples. It was found that the binding energies of C 1s, N 1s, and S 2p for the ZnS/SPI system were significantly shifted towards smaller binding energies compared to SPI and ZnS, which was attributed to the charge transfer that occurred between the interfaces. The displacement of the S 2p peak towards lower binding energy is mainly due to the electron energy transfer from SPI to ZnS in the composites, which leads to an increase in the electron density on the ZnS surface [40]. The analysis of the photoelectron spectra reveals a negative shift in C 1s and N 1s binding energies, indicating electronic redistribution effects associated with nitrogen vacancy formation within the composite system [27,28]. It has been shown that nitrogen vacancies can also enhance the photogenerated electron–hole separation and transport [41,42], thus enhancing their photocatalytic properties [29,30,31,32,33]. In the ZnS/SPI complexes, the binding energy of the Zn element was found to be shifted towards higher binding energies, indicating the presence of substantial interfacial electron coupling within the ZnS/SPI heterojunction system [25].

### 3.2. Optical and Electronic Properties

**Figure 6:**
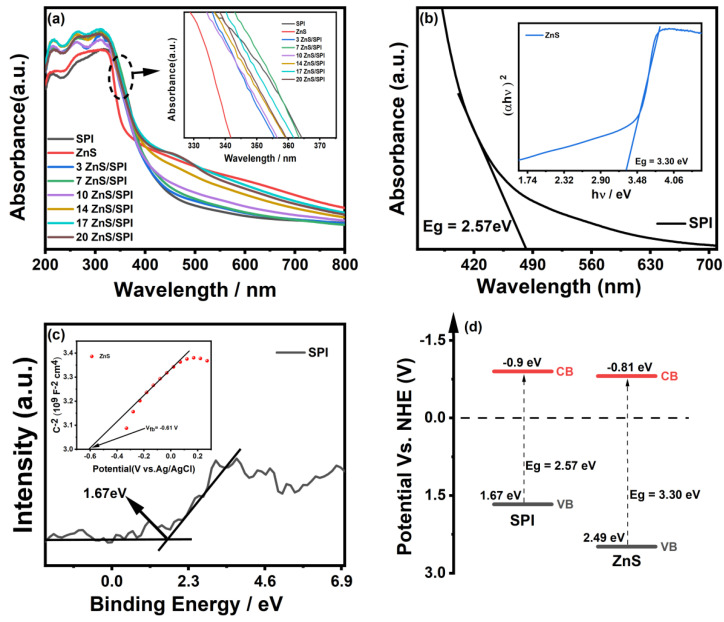
(**a**) UV−Vis diffuse reflectance spectra of SPI, ZnS, and ZnS/SPI. (**b**) Correspondence plot of (αhν)^2^ versus hν. (**c**) Mott−Schottky curve of ZnS and VBXPS of SPI. (**d**) The energy band structure diagram of SPI and ZnS.

Optical absorption characteristics of pristine SPI, pure ZnS, and the fabricated 14ZnS/SPI composite were examined through UV–visible diffuse reflection spectral analysis. In Figure 6a, it is obvious that SPI enhances the absorption of visible light by introducing ZnS. The intercept of the tangent line plotted on the wavelength axis from the UV–Vis spectrum of the sample shows that the SPI has an absorption band edge of 482 nm, which corresponds to a bandgap of 2.57 eV, and a relatively weak absorption of visible light [43]. Whereas the pure ZnS has an absorption band of 376 nm, which corresponds to a bandgap of 3.30 eV. It possesses a much wider absorption range of visible light, which is also consistent with what has been in the literature reports [25]. As can be seen in Figure 6a, the absorption bands of ZnS/SPI composites are significantly blue-shifted concerning pure SPI, which can be explained by the quantum size effect of ZnS nanorods [44,45]. Similarly, there is strong evidence that zinc sulfide is a nanoparticle. Such a modification facilitates the enhanced visible light harvesting capability while simultaneously promoting the spatial separation efficiency of photoinduced charge carriers.

The electronic structure calculations, performed using the Kubelka–Munk function, yielded fundamental bandgap energies. Spectroscopic evaluation (Figure 6b) established energy gaps of 3.30 eV for the ZnS phase and 2.57 eV for the SPI constituent. The Mott–Schottky curve was used to determine the potential (Efb) that ZnS can carry in a 0.5 M Na_2_SO_4_ electrolyte [46]. According to electrochemical analysis, the positive gradient of the Mott–Schottky plot demonstrates that the sample exhibits n-type semiconductor characteristics, whereas the negative gradient reveals p-type semiconductor properties [47]. The VBXPS spectrum of SPI and the Mott–Schottky curve of ZnS are shown in Figure 6c, which clearly shows that ZnS is an n-type semiconductor. The valence band potential of SPI is shown to be 1.67 eV and the conduction band potential of ZnS is shown to be −0.81 eV, respectively. ZnS as an n-type semiconductor, has a conduction band potential = flat band potential −0.2 V, where 0.2 is an empirical value and is not an exact value, i.e., the conduction band potential of ZnS = −0.61 − 0.2 eV = −0.81 eV [48]. Therefore, the conduction band of SPI is −0.9 eV as well as the valence band of ZnS is 2.49 eV can be calculated by the formula E_g_ = E_CB_ + E_VB_. According to the aforementioned experimental findings, the electronic band configuration of SPI and ZnS has been established, as illustrated in Figure 6d.

**Figure 7:**
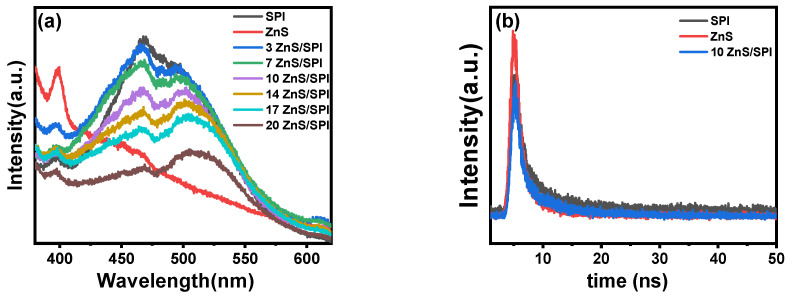
(**a**) Comparison of the photoluminescence spectra of SPI, ZnS, and ZnS/SPI composites; and (**b**) Transient PL decay spectra for pure SPI, ZnS, and 10ZnS/SPI samples.

When a photocatalyst is irradiated by a light source larger than the energy of its absorption band, it causes the electrons to jump from the ground state to the excited state, and this energy is released using fluorescence when the electrons return to the ground state from the excited orbital. Consequently, a decrease in photoluminescence emission intensity correlates with reduced recombination kinetics of photoexcited charge carriers. According to the aforementioned experimental observations, the interfacial charge carrier migration between the SPI and ZnS heterojunction appears to be crucial in determining the photocatalytic performance of the in situ synthesized ZnS nanocrystals, facilitating efficient charge separation at the interface. This mechanistic proposition was subsequently investigated through steady-state and time-resolved photoluminescence (PL) spectroscopic analysis. As shown in Figure 7a, it can be seen that SPI exhibits a strong emission peak centered at 475 nm, while ZnS has the weakest peak. Compared to the SPI, the intensity of ZnS/SPI composites at this emission peak is significantly reduced, indicating a significant enhancement in the charge carrier dissociation effectiveness under photoexcitation.

The carrier dynamics and charge separation behavior of the obtained samples were investigated through time-resolved luminescence spectroscopy (TRPL). The luminescence decay curves were quantitatively assessed via multi-exponential fitting analysis, as presented in Figure 7b, which revealed distinctive carrier lifetime characteristics among pristine SPI, ZnS, and the 10ZnS/SPI heterojunction composite. As shown in Table 1, the quantitative analysis demonstrated that the average carrier lifetime (<τ_avg_>) of pristine SPI and ZnS exhibited relatively rapid PL decay kinetics, yielding values of 5.21 ns and 3.38 ns, respectively. Significantly, the 10ZnS/SPI heterojunction manifested an extended carrier lifetime of 9.21 ns, attributable to the establishment of an efficient heterojunction interface between the ZnS and SPI components. Within this interfacial architecture, photogenerated electrons undergo facile transfer from the conduction band (CB) of ZnS to the valence band (VB) of SPI, thereby facilitating spatial charge separation and suppressing direct electron–hole recombination. A detailed analysis of the decay parameters revealed substantially reduced τ_1_ and τ_2_ values (1.06% and 4.93%, respectively) for the 10ZnS/SPI composite compared to its pristine counterparts, corroborating the enhanced charge separation efficiency at the heterojunction interface [49]. The optimized carrier dynamics manifested through prolonged carrier lifetimes and enhanced charge separation efficiency represents a crucial advancement for photocatalytic applications. In particular, the extended carrier lifetime facilitates more efficient redox reactions by providing sufficient temporal windows for charge transfer processes, consequently enhancing the overall photocatalytic efficiency [50]. These findings demonstrate that the rational construction of the ZnS/SPI heterojunction architecture effectively promotes carrier separation, offering valuable design principles for the development of high-performance polymer photocatalytic materials.

This enhanced charge carrier separation efficiency can be attributed to the transfer of photoexcited electrons from the valence to the conduction band via the ZnS/SPI interfacial junction, effectively suppressing the electron–hole recombination process [24]. It is shown that photo-excited electron transfer from SPI to ZnS nanoparticles is effective and can further improve the photocatalytic activity.

**Figure 8:**
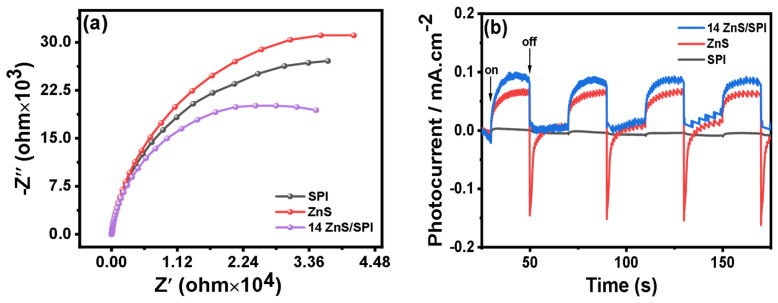
(**a**) EIS Nyquist plot of electrochemical impedance spectra; and (**b**) Photocurrent potential curves of SPI, ZnS, and 14ZnS/SPI composite electrodes in a 0.5 mol/L Na_2_SO_4_ aqueous solution.

To systematically evaluate the electrochemical characteristics of the as-synthesized samples, the interfacial charge transport behavior of SPI, ZnS, and 14ZnS/SPI samples was investigated using electrochemical impedance spectroscopy (EIS) under dark conditions. According to the Nyquist plots in Figure 8a, the semicircular arc dimensions reflect the interfacial impedance between electrode and electrolyte, where decreased arc diameters indicate reduced interfacial charge-transfer resistance [51]. The incorporation of ZnS nanostructures into the SPI matrix minimizes the interfacial charge-transfer impedance. Notably, the 14ZnS/SPI composite exhibited the most efficient electron transport kinetics, suggesting that the engineered ZnS/SPI heterojunction architecture facilitates enhanced charge carrier mobility across the interface. The photocarrier dynamics and charge separation capabilities were evaluated through photoelectrochemical measurements under periodic light on–off cycles, generating transient photocurrent responses (i–t curves) for pristine SPI, ZnS, and the 14ZnS/SPI heterostructure. The photoelectrochemical data presented in Figure 8b reveals superior photocurrent generation by the 14ZnS/SPI heterojunction system, whereas pristine SPI exhibited minimal photoresponse under identical illumination conditions. This increase in photocurrent intensity is further evidenced by the fact that 14ZnS/SPI composites show higher light utilization as well as more efficient electron transfer. This phenomenon primarily originates from the improved optical response in the visible spectrum range by Zn vacancies in ZnS, which promotes photogenerated electron–hole separation [24]. In addition, the ZnS/SPI heterojunction structure also favors the charge transfer between ZnS and SPI.

### 3.3. Photocatalytic Activity and Mechanism

**Figure 9:**
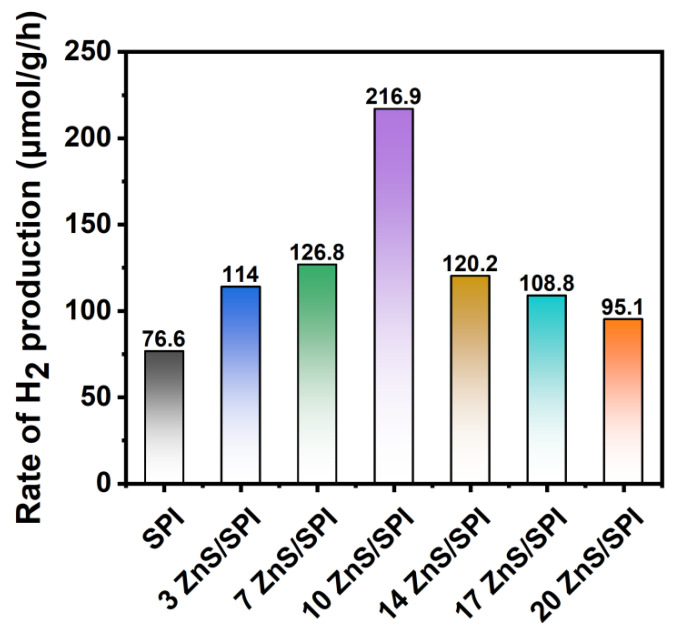
The corresponding photocatalytic hydrogen production rates of SPI, ZnS, and ZnS/SPI composite materials under solar irradiation (λ > 300 nm, 10 vol% aq. triethanolamine).

To examine the influence of surface-immobilized ZnS nanostructures, synthesized through in situ crystallization on SPI matrix, on the photocatalytic efficiency, different catalysts were tested for their photocatalytic hydrogen production activity under fully irradiated light using triethanolamine (TEOA) as a hole sacrificial agent. According to the previous literature, Zn vacancies are the key factor in improving the performance of visible light photolysis for hydrogen production from water [19]. The experimental results show that the photolytic water hydrogen production performance of the ZnS/SPI composite system is significantly better than that of SPI, as shown in Figure 9. It reaches the maximum value in the 10ZnS/SPI system, and then shows a decreasing trend. The relatively low H_2_ generation rate of 76.6 µmol/g/h for pure SPI is attributed to its limited absorption of visible light, poor charge transport ability, and small specific surface area [43]. The corresponding hydrogen production rates for 3ZnS/SPI, 7ZnS/SPI, 10ZnS/SPI, 14ZnS/SPI, 17ZnS/SPI, and 20ZnS/SPI were 114 µmol/g/h, 126.8 µmol/g/h, 216.9 µmol/g/h, 120.2 µmol/g/h, 108.8 µmol/g/h, and 95.1 µmol/g/h. The maximum hydrogen production rate was achieved on 10ZnS/SPI, which was about 2.8 times higher than the hydrogen production rate of pure SPI. The enhanced photocatalytic H_2_ evolution efficiency can be rationalized by the synergistic effects of increased visible-light harvesting capability and accelerated charge carrier separation, originating from the zinc-deficient sites within the ZnS lattice [19]. Meanwhile, the formation of the Z-type heterojunction structure of ZnS/SPI was utilized to promote the charge transfer at the interfaces between ZnS and SPI. Consequently, the enhanced photocatalytic efficiency manifests in accelerated H_2_ evolution kinetics, demonstrating substantially superior catalytic activity compared to pristine SPI.

**Figure 10:**
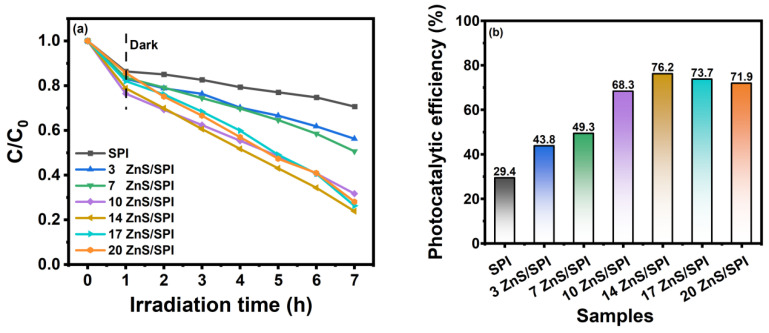
(**a**) The comparison of the photocatalytic degradation rate of SPI and ZnS/SPI on MO under light (λ > 420 nm); and (**b**) Photocatalytic degradation efficiency of SPI and ZnS/SPI composites.

Considering the fact that methyl orange (MO) is a widespread organic contaminant, its good antioxidant and photolysis resistance make it a typical pollutant. Therefore, it frequently serves as a model system in photocatalytic degradation studies [52]. Therefore, to further evaluate the photocatalytic activity of SPI and ZnS/SPI composites, the photocatalytic performance of SPI and different ratios of ZnS/SPI composites were evaluated with the target of degrading 40 mg/L MO aqueous solution. From Figure 10a, it can be seen that the photocatalytic degradation activity is much higher in the xenon lamp simulated sunlight-driven compared to the dark case. Such a result suggests that the degradation of MO is indeed accomplished by the sunlight-driven catalyst. The results from Figure 10a show that the photocatalytic degradation activity of ZnS/SPI composites is gradually enhanced with the increase in ZnS content. And, the highest photocatalytic degradation activity of the 14ZnS/SPI composites was observed, and the degradation rate was 76.2%. Beyond this point, the continuous elevation of ZnS loading in the ZnS/SPI hybrid system resulted in a gradual decline of its photocatalytic efficiency towards pollutant decomposition. The primary reason lies in the suppression of solar light harvesting and photo-induced charge carrier generation caused by superfluous ZnS, resulting in reduced photocatalytic efficiency. [53]. Moreover, Figure 10b illustrates the comparative photocatalytic performance of SPI and ZnS/SPI composites towards MO degradation. The results demonstrate that all composite materials exhibited enhanced photocatalytic activity compared to pristine SPI. Notably, the 14ZnS/SPI composite displayed the most efficient degradation capability, achieving 2.6-fold higher activity than pure SPI.

**Figure 11:**
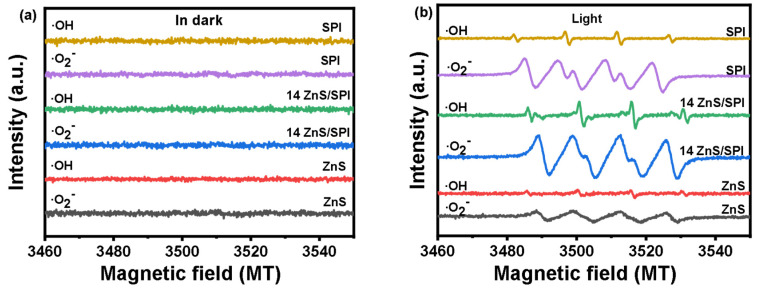
ESR spin trapping spectra of SPI, ZnS, and 14ZnS/SPI composites: (**a**) In dark conditions; and (**b**) Under lighting conditions.

Understanding the fundamental principles underlying photocatalytic processes plays a crucial role in advancing novel photocatalytic material design. In the photocatalytic process, the photo-induced holes (h^+^) participated in oxidation reactions with H_2_O/OH^−^ species to yield hydroxyl radicals (·OH), whereas the excited electrons (e^−^) undergo reduction reactions with molecular oxygen (O_2_) to produce superoxide radical species (·O_2_^−^). Both and ·O_2_^−^) generated by redox reactions are key factors affecting the photocatalytic activity, which is important in photocatalytic degradation as well as hydrogen production from photolyzed water. Therefore, the SPI, ZnS, and 14ZnS/SPI samples were tested using ESR to gain insights into the transport and separation mechanisms of photogenerated carriers. The electron spin resonance (ESR) signals were measured for the synthesized specimens in H_2_O (for DMPO-·OH) and CH_3_OH (for DMPO-·O_2_^−^) solutions, employing 5,5-dimethyl-1-pyrroline-N-oxide (DMPO) as the radical trapping agent [54]. Figure 11a demonstrates the absence of DMPO-·OH and DMPO-·O_2_^−^ radical signals in SPI, ZnS, and 14ZnS/SPI samples without illumination. Upon light irradiation (Figure 11b), ESR analysis reveals intense **·**O_2_^−^ signals accompanied by weak ·OH peaks for SPI, while ZnS exhibits minimal **·**O_2_^−^ intensity with negligible ·OH radical detection. The reason for this is that SPI has a high conduction band position (−0.9 eV) and ZnS has a low valence band position (2.49 eV), which gives it better reduction and oxidation capabilities, respectively. However, the weak ·OH radical detection signal of ZnS may be due to the presence of defect states or adsorbed impurities on the surface of ZnS. These defect states can trap photogenerated carriers and change their migration path and reaction direction. Interestingly, the 14ZnS/SPI composite shows the significant enhancement of both ·O_2_^−^ and ·OH signals, a phenomenon which can be primarily explained by the interfacial heterojunction architecture established between SPI and ZnS through robust molecular interactions. These experimental observations align well with the electronic band structures of the SPI and ZnS components.

**Figure 12:**
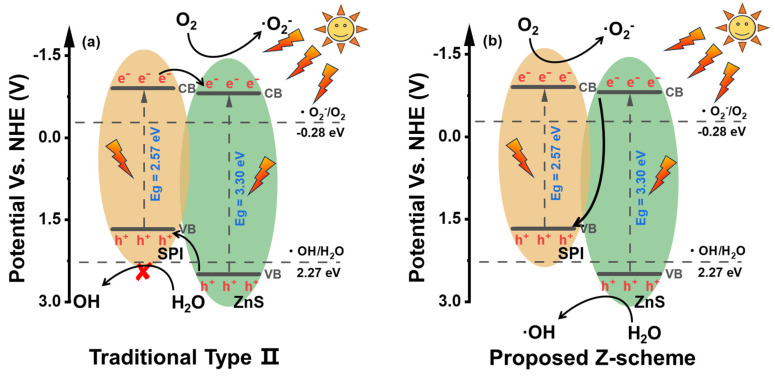
Schematic illustration of (**a**) traditional type−II heterojunction; and (**b**) direct Z−scheme charge transfer mechanism for ZnS/SPI system.

The rational design and fabrication of the ZnS/SPI heterojunction system demonstrate significant enhancement in photocatalytic performance through a direct Z-scheme charge transfer mechanism. The advantages of the Z-type heterostructured photocatalysts are that they can effectively improve the separation efficiency of photogenerated electrons and holes while maintaining the stronger reducing capacity of electrons in the conductive band of the reducing photocatalyst and the oxidation capacity of holes in the valence band of the oxidizing catalyst [55]. The quantitative analysis reveals that the optimized 14ZnS/SPI composite exhibits superior photocatalytic activity. The improved photocatalytic activity originates from the optimized energy level configuration and facilitated electron–hole pair transport channels. Electron spin resonance (ESR) spectroscopy provides definitive evidence for the Z-scheme mechanism, wherein strong DMPO-·O_2_^−^ signals were detected on the surface of SPI and intense DMPO-·OH signals were observed on the surface of ZnS under light irradiation. If it is a type-II heterojunction between SPI and ZnS (in Figure 12a), the holes of ZnS should transfer to the VB of SPI, and the electrons of SPI should transfer to the CB of ZnS, which will lead to the weakening of the ·OH and ·O_2_^−^ detection signals of ZnS/SPI composites, respectively. However, both the ·OH and ·O_2_^−^ signals of the ZnS/SPI composite were enhanced, indicating that it is a Z-scheme heterojunction between SPI and ZnS (in Figure 12b). The Z-scheme configuration effectively maintains the strong reduction potential of SPI for O_2_ reduction (generating ·O_2_^−^) and the robust oxidation capability of ZnS for H_2_O oxidation (producing ·OH), thereby optimizing the system’s overall redox efficiency. This mechanistic interpretation is further corroborated by the enhanced photocurrent response and decreased charge transfer resistance observed in electrochemical measurements. The synergistic integration of these components through the Z-scheme pathway not only facilitates more efficient charge carrier dynamics but also preserves the intrinsic redox potentials of both semiconductors, representing a significant advancement in heterojunction photocatalyst design.

## 4. Conclusions

To conclude, an innovative straightforward Z-type ZnS/SPI composite photocatalyst was prepared by in situ crystalline growth of ZnS nanoparticles on the surface of SPI. The constructed ZnS/SPI Z-type heterojunction with broad absorption in the visible region effectively promotes the separation and transfer of photogenerated electron–hole pairs at the close contact interface between ZnS nanoparticles and SPI. Owing to the mentioned beneficial features, experimental measurements demonstrated that the 10ZnS/SPI hybrid material achieved the maximum photocatalytic hydrogen production rate of 216.9 µmol/g/h when subjected to complete illumination, surpassing the pristine SPI (76.6 µmol/g/h) by approximately 2.8 times. The synergistic effect of the ZnS/SPI heterojunction structure is the main reason for the enhanced photocatalytic performance. Herein, we demonstrate a facile and cost-effective approach for developing highly efficient π-conjugated polymer-based photocatalysts with enhanced photocatalytic performance.

## Figures and Tables

**Table 1:** Time-resolved fluorescence decay parameters of SPI, ZnS, and 10 ZnS/SPI samples.

Samples%	A_1_ (%)	τ_1_ (ns)	A_2_ (%)	τ_2_ (ns)	A_3_ (%)	τ_3_ (ns)	<τ_avg_> (ns)
SPI	204.13	1.66	53.95	7.99	/	/	5.21
ZnS	191.80	1.45	216.77	1.45	22.32	9.03	3.38
10 ZnS/SPI	187.57	1.06	64.61	4.93	10.67	21.95	9.21

## Data Availability

The data presented in this study are available upon request from the corresponding author.

## Supplementary Materials

The following supporting information can be downloaded at: https://www.mdpi.com/article/10.3390/polym17050575/s1, Table S1: The amounts of the reactants for the synthesis of the ZnS/SPI samples.
