# Supplementary material for: In Situ Crystalline Growth ZnS Nanoparticles on Conjugated Polymer for Enhancement of the Photocatalytic Performance
Source: Polymers (Basel). 2025 Feb 21;17(5):575. doi: 10.3390/polym17050575 (PMC11902180; doi:10.3390/polym17050575)
Supplement: Supplementary file 1 [file polymers-17-00575-s001.zip › polymers-3471003-supplementary.pdf]

In situ crystalline growth ZnS nanoparticles on conjugated polymer for enhancement of the photocatalytic performance

Baotong Liu, Xuelian Li, Long Zhang, Chenghai Ma*, Ying Chen, Xinyu Wang, Hongli Wei, Pengfei Wang

School of Chemical Engineering, Qinghai University, Xining, 810016, China

Table S1. The amounts of the reactants for synthesis of the ZnS/SPI samples

Samples*	SPI	$Zn(CH_3COO)_2 \cdot 2H_2O$	C_2H_5NS	PVP	Synthetic yield
3 ZnS/SPI	0.970g	0.0676g	0.0462g	0.0042g	0.9528g
7 ZnS/SPI	0.930g	0.1576g	0.1079g	0.0099g	0.9488g
10 ZnS/SPI	0.900g	0.2252g	0.1541g	0.0142g	0.9653g
14 ZnS/SPI	0.860g	0.3153g	0.2158g	0.0198g	0.9218g
17 ZnS/SPI	0.830g	0.3828g	0.2621g	0.0241g	0.9559g
20 ZnS/SPI	0.800g	0.4504g	0.3083g	0.0283g	0.9374g

*All samples were synthesized by a facile immersion-hydrothermal method, heating at 180 ℃ for 12 hours. The amounts of the reactants were calculated according to the synthesis of 1 g sample.
